# Environmental enrichment protects against functional deficits caused by traumatic brain injury

**DOI:** 10.3389/fnbeh.2013.00044

**Published:** 2013-05-21

**Authors:** Erica M. Johnson, Kyle L. Traver, Stuart W. Hoffman, Catherine R. Harrison, James P. Herman

**Affiliations:** ^1^Department of Psychiatry and Behavioral Neuroscience, University of CincinnatiCincinnati, OH, USA; ^2^711th Human Performance Wing, Air Force Research Laboratory, Wright Patterson AFBOH, USA; ^3^Office of Research and Development, U.S. Department of Veteran AffairsWashington, DC, USA; ^4^Federal Aviation AdministrationWashington, DC, USA

**Keywords:** environmental enrichment, traumatic brain injury, morris water maze, controlled cortical impact, sensory neglect

## Abstract

Environmental enrichment (EE) increases cortical weight, neuronal density, dendritic branching, and angiogenesis, all of which may be critical for functional recovery following insult. Our study was designed to determine possible benefits of pre-exposure to EE in preventing functional deficits following traumatic brain injury (TBI) to the prefrontal cortex. To examine the benefit of EE, adult male rats were placed in an enriched environment for 15 days. Enrichment was provided through social interaction, exercise, olfactory stimulation, and new objects/toys to explore. Following enrichment, experimental and age-matched controls were subjected to a moderate medial prefrontal cortex injury via controlled cortical impact (CCI). After 1 week recovery, animals were behaviorally tested to assess memory, anxiety, and sensory neglect. Lesion-induced deficits in spatial memory [Morris water maze (MWM)] were significantly attenuated in EE pre-exposed rats 18–21 days following injury. In addition, TBI-induced sensory neglect was significantly reduced in EE rats relative to non-enriched animals. No differences in anxiety-like behavior on the elevated plus maze (EPM) were detected. The behavioral data suggest that EE is neuroprotective when applied prior to TBI, resulting in improved recovery following injury.

## Introduction

Environmental enrichment (EE), the provision of a rich and stimulating environment, induces neuronal changes that can ameliorate functional deficits associated with various degenerative diseases and injury (Horner and Gage, 2000; van Dellen et al., 2000; Passineau et al., 2001; Will et al., 2004; Gaulke et al., 2005). Early studies in rodents indicate that EE increases total brain and cortical weight (Henderson, 1970; Rosenzweig and Bennett, 1972; Rosenzweig et al., 1972). Subsequent studies document additional benefits of EE including increased neuronal density (Kempermann et al., 1997), dendritic branching (Greenough and Volkmar, 1973), cortical tissue synapses (Turner and Greenough, 1985), neuronal transmission (Rampon et al., 2000), and enhanced neurotrophic growth factor expression (Young et al., 1999; Ickes et al., 2000). In addition, EE regulates the hypothalamic-pituitary-adrenal (HPA) axis, mediating the release of glucocorticoids (GCs) such as corticosterone. The EE-induced hormonal and neurostructural changes are associated with improvements in memory (Chamove, 1989; Nilsson et al., 1999; van Praag et al., 2000; Schrijver et al., 2002; Benaroya-Milshtein et al., 2004) and reduced anxiety-like behaviors (Larsson et al., 2002).

The beneficial effects of EE on cortical structure and function support its use as a therapy to improve recovery from traumatic brain injury (TBI) (Will et al., 1977, 2004; Horner and Gage, 2000; Passineau et al., 2001; Chen et al., 2005; Gaulke et al., 2005; Giza et al., 2005; Wagner et al., 2005; Kline et al., 2007). Clinical symptoms of TBI include anxiety (Hiott and Labbate, 2002), cognitive and memory deficits (Levin et al., 1988), sensory impairments (Lew et al., 2012), and attentional impairments (Willmott et al., 2009). Cortical and subcortical lesions in regions such as the hippocampus occur most frequently following TBI in the frontal lobe (McDowell et al., 1997), leading to lasting working memory deficits (Fox et al., 1998), prominent anxiety (Jorge et al., 2004), and executive dysfunction (Eslinger, 1996). The neurological and behavioral effects of EE reduce TBI-induced deficits and improve recovery time (Will et al., 1977, 2004; Passineau et al., 2001; Chen et al., 2005; Gaulke et al., 2005; Giza et al., 2005; Wagner et al., 2005; Kline et al., 2007). Importantly, the cognitive benefits of EE are also demonstrated by a reduced deficit in memory performance following brain injury (Hamm et al., 1996; Will et al., 2004; Komitova et al., 2005).

Post-TBI EE therapy is effective in reducing negative outcomes, but its efficacy is limited by the fact that damage has already occurred, and in this usage is palliative, rather than preventative manipulation. Applying enrichment *before* injury may afford additional resiliency against TBI-induced deficits through modulation of various endogenous brain responses to injury that have deleterious effects on functional outcome. While the studies above reflect the neuroanatomical and behavioral effects of EE applied following TBI, little research exists exploring the use of EE as a prophylactic measure on adult animals. Kozlowski and colleagues evaluated the effect of EE on immature rats before controlled cortical impact (CCI) and found that pre-TBI EE paradoxically increased lesion size but ultimately enhanced recovery as indicated by shortened motor coordination recovery time (Kozlowski et al., 2004). A recent study with adult rats by Lanosa and colleagues demonstrated that pre-injury exposure to EE enhanced the amount of astrocyte involvement in the formation of new synapses following injury (Lanosa et al., 2011). However, these studies did not examine the resiliency effects of EE against cognitive deficits.

Positive effects of EE on numerous indices of neuronal health and viability suggest that exposure to enrichment may render the brain resistant to deleterious effects of a subsequent insult. Thus, pre-exposure to EE may comprise a neuroprotective strategy to reduce the negative impact of TBI behavioral deficits in memory, sensory neglect, and anxiety when applied prior to injury. If neuroprotective, directed use of EE may be a viable training mechanism to improve resiliency against the consequences of physical brain trauma. The present experiment sought to tests the efficacy of prior EE in limiting the negative consequence of TBI on sensory, cognitive and emotional functions. To this end, adult male rats were placed in an enriched environment prior-to receiving a moderate medial prefrontal cortex injury via CCI. Our data suggest that prophylactic EE may effectively reduce the impact of TBI on sensory impairment and memory deficits.

## Materials and methods

### Animals

Forty-eight male Sprague-Dawley rats (Charles River Laboratory, Wilmington, MA) were used in this study. Rats were approximately 120 days (400–500 g) of age at the commencement of experimentation. The animals were housed in a room with controlled temperature and a 12 h light/dark cycle (lights on at 0600) with *ad-libitum* access to food and water. All experimental protocols were approved by the Wright Patterson Air Force Base Institutional Animal Care and Use Committee, and conformed to the guidelines published in the National Institutes of Health (NIH) Guide for the Care and Use of Laboratory Animals.

### Housing conditions

**Figure 1:**
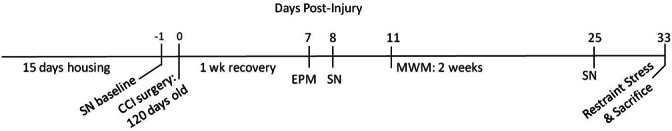
**Experimental timeline.** The experiment consisted of 15 days of housing (either enriched or standard) followed by controlled cortical impact (CCI) surgery. After 1 week of recovery, the following behavioral tests were conducted: sensory neglect (SN), elevated plus maze (EPM), and Morris water maze (MWM).

Two housing conditions were used: environmentally enriched condition (EC) and standard control (ST), both for 15 days before injury (see Figure 1 for experimental timeline). The ST group was housed in standard-sized polycarbonate rat cages, two per cage. Animals in both housing conditions were subjected to a medial prefrontal TBI via CCI. An additional group of animals received sham injury and were maintained under standard housing conditions. Sixteen animals were placed in each cohort: EC-TBI, ST-TBI, and Sham.

**Figure 2:**
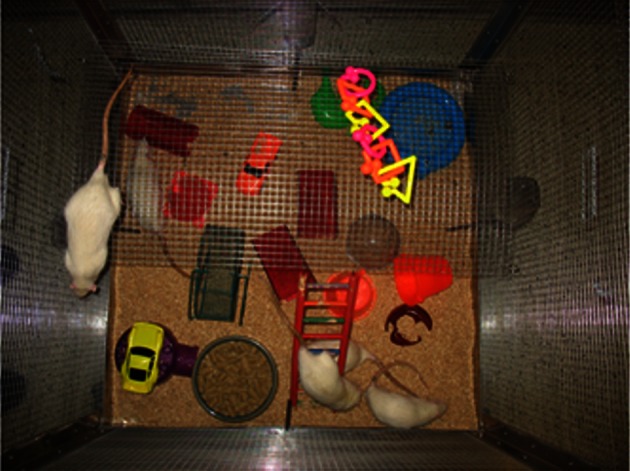
**Enriched housing condition (EC group).** The EC group was housed in large wire mesh cages (1 m^3^) with levels and access to running wheels and toys, replaced twice weekly.

The EC group was housed eight animals per cage in large wire mesh cages (1 m^3^) with two levels and access to running wheels and toys (e.g., ropes, plastic cars and trucks, plastic tunnels, ladders and running wheels) (Briones et al., 2004; Lippert-Gruener et al., 2007) (Figure 2). The objects inside the EC cages were replaced by new toys twice weekly (Briones et al., 2004) in order to provide the animals with opportunities for learning and sensory stimulation. In addition, twice a day each EC animal underwent individual motor skills training (MST), an enriching activity that involves learning a complex motor task and combines coordination, balance, concentration, and learning components. MST consisted of an elevated obstacle course of parallel bars, balance beams, ropes, ladders, and chains (Jones et al., 1999). Animals were trained on the MST course between 1300 and 1500. EC animals were also exposed to daily olfactory stimulation consisting of variously perfumed paper strips presented in the cage for 10 min (Maegele et al., 2005). The scents used each day were chosen randomly and consisted of peppermint, lavender, or cinnamon. ST-TBI and Sham animals were handled daily, yet otherwise left undisturbed in their group cages without any additional stimulating enrichment.

### Controlled cortical impact

After 15 days of housing in either EC or ST conditions, animals were subjected to TBI in the prefrontal cortex by means of CCI. This region in the rat is homologous to the anterior cingulate cortex in humans, implicated in frontal lobe syndrome (Leonard, 1969). The CCI model of brain injury in rats results in pathophysiology similar to human TBI including contusion and axonal injury (Dixon et al., 1996). Animals were anesthetized with isoflurane (5% induction, 2% maintenance) and mounted in a stereotaxic apparatus with heads fixed in a horizontal position. Blood oxygen saturation (SpO_2_) was monitored and maintained at levels >90% and body temperature was maintained at 37°C with a homeothermic heating blanket system. A midline incision was made and the scalp and fascia were retracted to expose the cranium. Using a 6 mm diameter trephan, a craniectomy was made over the medial frontal cortex, 3 mm anterior to bregma. Once the dura was exposed, a bilateral contusion was induced using a 5 mm diameter anvil with an electric contusion impactor (Custom Design and Fabrication, Virginia Commonwealth University Medical Center, Richmond, Virginia). The injury was made with a velocity of 2.25 m/s, depth of 3 mm, and 50 ms of brain contact. Bleeding was stopped with gauze and cool saline, and the scalp was subsequently sutured with 7 mm surgical staples. Sham animals received craniectomy but no cortical impact. Following recovery from anesthesia, animals were returned to their original cages with cage mates. The EC animals were returned to their original cages, but no longer received additional enrichment, and the levels and toys were removed from the cages. The animals recovered for 1 week before the commencement of behavioral tests.

### Sensory neglect

The sensory neglect test is used to observe responsivity to focal somatosensory stimuli (Hoffman et al., 2003). All animals were tested for sensory neglect at baseline and post-injury. Baseline measurements of sensory neglect were taken one day prior to CCI or sham surgery. Further testing was conducted at 8 and 25 days post-surgery. The test consisted of a 2 cm diameter sticker being placed on the distal-radial area of both forelimbs. The animal was then placed in a clear empty shoebox cage and the latency to remove both stickers was recorded. Each animal received 3 trials with approximately 5 min intervals. The maximum length of a trial was 2 min, at which time any remaining stickers were removed.

### Elevated plus maze

The elevated plus maze (EPM) is a standard test of anxiety-like behaviors in rodents (Benaroya-Milshtein et al., 2004). All animals were tested at day 7 post-injury. Animals were placed in the center of the maze, consisting of two open (10.2 × 102 cm) and two enclosed arms (10.2 × 102 × 30.5 cm), elevated 53.3 cm above the floor. At the start of the test, animals were placed facing one of the enclosed arms. Each animal was tested for one 5 min trial and the maze was cleaned with 70% ethanol after each trial. Ethovision software was used to record the behavior of the animal and time spent in each area of the maze. Behaviors which were recorded included rearing, grooming, and head dipping.

### Morris water maze

The Morris water maze (MWM) is an established test of spatial learning and memory (Buccafusco, 2009). All animals were tested on the MWM beginning 11 days post-surgery. Animals were placed in a 178 cm diameter dark circular tank filled with water (approximately 37 cm deep). The tank was divided evenly into four quadrants (A, B, C, D) with appropriate marking of the quadrants on the top edge of the tank. A 10.2 cm diameter clear plexiglass platform was submerged to a depth of 2 cm below waterline and placed approximately 28 cm from the wall of the pool in quadrant C. Latency to find the hidden platform was recorded using Ethovision software that tracked and timed the animals in the tank until they reached the platform. The position of the platform remained the same throughout the experiment.

The MWM consisted of 10 tests performed over 12 days (two 5-day blocks with a 2-day break between the blocks to test memory retention). Days 1–11 consisted of acquisition testing and were comprised of 2 trials per day. For each trial, the animal was placed in the pool at random quadrants, facing the wall. If the animal did not find the platform in 90 s, they were physically guided to it. Upon reaching the platform the animal remained there for 10 s and then was removed for a 30 s interval before the start of the second trial. MWM day 12 consisted of only one probe trial with the platform removed and time spent in quadrant C was recorded.

### Histology

Following the behavioral tests and stress challenge, animals were briefly anesthetized with 5% isofluorane and decapitated via guillotine. The brains from half of each group were removed and split in half sagittally (eight brains in each cohort: EC-TBI, ST-TBI, and Sham). The remaining brains were taken for biochemical determinations, however, freezer failure resulting in the loss of approximately half of the samples compromised our ability to perform biochemical and more detailed histolgical analyses that had been planned. Coronal sections of the intact brain hemispheres (14 μm) through the prefrontal cortex and hippocampus were cut on a cryostat and stored at −20°C. Every fourth section was used for lesion analysis using Nissl staining to identify cell bodies and grossly visualize damage from the CCI. Slides were fixed in 4% paraformaldehyde for 20 min. After being rinsed in 1× phosphate buffered saline (PBS) and dried overnight, slides were placed in cresyl violet for 4–5 min. Racks were rinsed 5 times with H_2_O and dehydrated using a variety of alcohol concentrations and xylenes. The slides were coverslipped and imaged using an Axio Imager.Z1 (Carl Zeiss Microimaging, Thornwood, NY). The Nissl-stained coronal sections of the prefrontal cortex were visually analyzed for gross differences in lesion size. In both ST-TBI and EC-TBI animals, there was substantial loss of Nissl neuronal profiles in the dorsal prefrontal cortex, typically compromising the anterior cingulate cortex, with occasional damage seen in the prelimbic cortex. Qualitatively, the extent of cortical damage was similar between the two groups. Due to use of unfixed tissue, we were unable to perform detailed quantitative mapping of the extent of prefrontal cortex damage. Note that prefrontal cortical damage produced by this CCI approach has been previously documented (Hoffman et al., 2003).

### Statistical analysis

All results are expressed as mean ± standard error (SE), unless depicted differently in the figures. The data were tested for normality and equal variance using the Kolmogorow-Smirnov test and *F*-test, respectively. Outliers for each data set were identified using Grubb's test with α = 0.05 (Barnett and Lewis, 1994). The significance level for all tests was 0.05. A One-Way analysis of variance was used to compare groups ST-TBI, EC-TBI, and Sham. If day was included as a factor, a mixed-design ANOVA was used. Since the specific hypotheses tests were identified a priori, planned comparisons were performed regardless of the outcome of the ANOVA. *Post-hoc* paired comparisons of groups used Tukey simultaneous confidence intervals with a 0.05 experimentwise error level.

## Results

Four rats were excluded from all analyses (1 ST-TBI, and 3 Sham) due to death or poor post-surgical recovery, resulting in the following sample sizes for behavioral analysis: 16 EC-TBI, 15 ST-TBI, and 13 Sham.

### Sensory neglect

**Figure 3:**
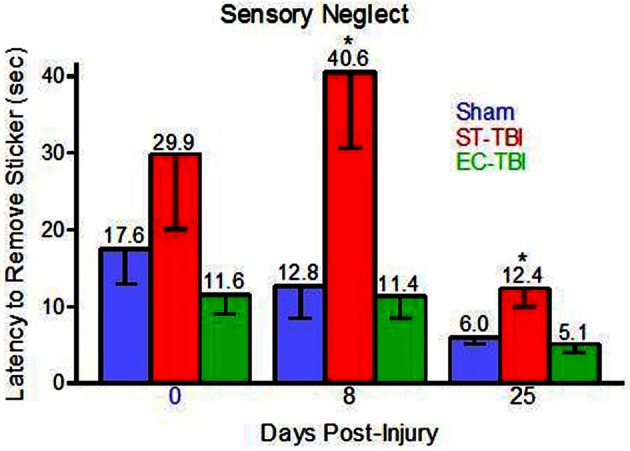
**Reduced sensory neglect deficit associated with enrichment before injury.** Sham and EC-TBI animals removed the stickers significantly faster than the ST-TBI group at days 8 and 25 post-injury. Data shown are Mean − SE. ^*^Significantly different than Sham and EC-TBI using Tukey's *post-hoc* test.

One rat (Sham) was removed from all analyses for chewing the sticker instead of trying to remove it. Prior to TBI, there was not a significant effect of group on latency to sticker removal, indicating that housing conditions did not affect sensory neglect. Following TBI, there were significant effects of group on sticker removal latency on day 8 [*F*_(2, 40)_ = 6.33, *p* < 0.01] and day 25 [*F*_(2, 40)_ = 6.13, *p* < 0.01]. *Post-hoc* analysis indicated that both Sham and EC-TBI removed the stickers significantly faster than the ST-TBI group at both post-injury time points (Figure 3). A mixed design ANOVA using group and day as factors did not have a significant group/day interaction. A scatter graph of individual rats indicated an elevated mean for the ST-TBI group at baseline was due to the contribution of only two rats. Since mean differences during baseline were not considered meaningful, it was concluded that housing conditions did not affect sensory neglect and TBI-induced sensory neglect deficits were attenuated when enrichment was applied prior to injury, particularly on day 8.

### Anxiety-like behavior: elevated plus maze

**Figure 4:**
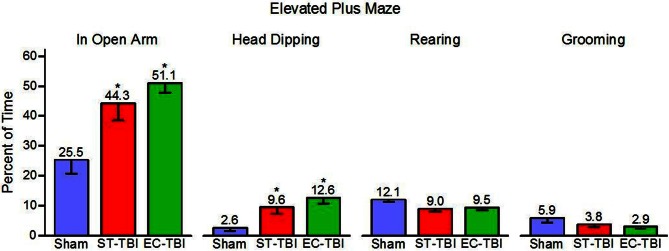
**Risk-taking on the elevated plus maze after injury is not remedied by enrichment.** ST-TBI and EC-TBI animals spent more time exploring the open arms and head dipping than Sham animals. No significant differences in rearing or grooming were observed. Data shown are Mean − SE. ^*^Significantly different than Sham and EC-TBI using Tukey's *post-hoc* test.

Percent time in the open arm, head dipping, rearing, and grooming were tested as indicators of anxiety-related behaviors in the EPM [One-Way analysis of variance (ANOVA)]. There were significant effects of groups on open arm time [*F*_(2, 40)_ = 8.08, *p* < 0.01] and head dipping [*F*_(2, 40)_ = 7.23, *p* < 0.01], but no significant group effects on rearing or grooming (Figure 4). *Post-hoc* paired comparisons indicated that both ST-TBI and EC-TBI groups spent more time in the open arm and more time head dipping than the Sham group. Time spent in the open arm and head dipping can be associated with reduced anxiety-like behavior. However, it is important to note that prefrontal damage can increase risk-taking behavior (Bechara et al., 1994; Floden et al., 2008) which may result in increased open arm time via a non-anxiety related mechanism (Pandey et al., 2009).

### Spatial memory: morris water maze

**Figure 5:**
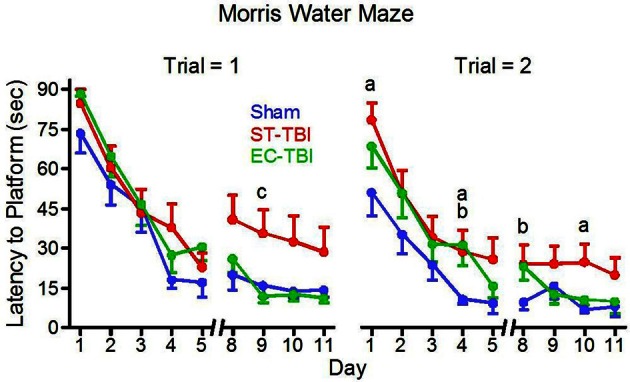
**Reduced deficits on the morris water maze task associated with enrichment before injury.** EC-TBI and Sham animals reached the hidden platform significantly faster than ST-TBI animals in trial 1 following 2 day break for memory retention. ^a^*p* < 0.05 Sham vs. ST-TBI, ^b^*p* < 0.05 Sham vs. EC-TBI, ^c^*p* < 0.05 ST-TBI vs. EC-TBI using two-tailed 2-sample *t*-test.

Five separate trials throughout the MWM testing were considered outliers and removed from analysis (1 Sham, 1 ST-TBI, 3 EC-TBI). A mixed-design ANOVA on latency to reach the submerged platform was performed for each trial separately using days 8–11, following a two-day break when animals were not tested. For trial 1 there was a significant difference among the groups [*F*_(2, 41)_ = 4.56, *p* = 0.01]. *Post-hoc* analysis indicated that in trial 1, following the 2-day break, both Sham and EC-TBI located the platform significantly faster than the ST-TBI group (Figure 5). For trial 2 there was not a significant difference among the groups. The EC-TBI animals performed similarly to the Sham controls, demonstrating no injury-induced deficits in spatial memory. ST-TBI animals, however, demonstrated impaired MWM performance, especially during trial 1 after the two-day break, indicative of memory recall deficits.

No significant differences in swim speed (range = 25.3 ± 1.1 to 28.0 ± 1.1 cm/s) were observed among the groups on the first or final day, confirming that the differences observed in escape latencies could not be attributed to motor deficits affecting swimming speed. On the last day of MWM testing, the platform (originally in quadrant C) was removed and time spent in quadrant C was analyzed for memory retention. A One-Way ANOVA revealed no significant difference among the groups for time in quadrant or distance swam.

## Discussion

The results of the present study provide evidence that EE *before* experimental TBI results in sparing of cortical function. Approximately 2 weeks of enrichment prior to injury in adult animals blocked the deleterious effects of TBI on spatial memory and sensory discrimination, indicated by performances indistinguishable from the Sham group in the MWM and sensory neglect test. Enriched animals therefore had a better prognosis following experimental TBI. This is likely caused by long-term effects of EE on neuroplastic responses to challenge.

Findings from the MWM indicated that EE blocks the development of spatial memory deficits due to subsequent frontal lobe injury. Because the animal relies solely on spatial cues and memory, decreased latency to the platform is indicative of improved spatial memory. The fact that MWM swim speeds were similar among the groups indicates that there were no motor deficits associated with injury or treatments, and thus impaired MWM performance in the ST-TBI group can be interpreted to represent memory deficits associated with prefrontal cortex lesions. TBI did not impair the acquisition of the MWM task during the first five days as indicated by similar latencies among all groups. In days 8–11, however, ST-TBI animals showed increased latency to find the platform on the first trial. Trial 1 is the first test of each day and is an indication of memory recall, and increased latency on this trial is indicative of functional impairment. Enriched injured animals performed similarly to the Sham controls, indicating that EE prior to injury blocks the deleterious effects of TBI.

The two-day break following test day five provided for an additional test of memory recall. ST-TBI animals performed significantly worse in trial 1 after the two-day break, whereas EC-TBI and Sham animals continued to improve time to platform even after the break, reflective of long-term memory retention. It can therefore be concluded from the MWM results that injury to the prefrontal cortex results in memory recall deficits that can be prevented by applying EE before injury.

The sensory neglect task revealed significant impairments resulting from injury to the prefrontal cortex, which was abolished by pre-enrichment. The sensory neglect task is a good indicator of responsiveness to focal somatosensory stimuli (Hoffman et al., 2003). The enhanced EC-TBI performance after injury could be indicative of improved attention and sensitivity. Overall, the improvements observed in the MWM and sensory neglect test indicate that enrichment positively affects structures distal to the site of injury, including the hippocampus and sensory fields. Our data suggests that these structures were made resilient to injury through the enrichment training paradigm and therefore protected from the deleterious effects of TBI.

The EPM is used frequently to test for anxiety-like behavior, but in this experiment it revealed interesting results about the effect of frontal injury on risk-taking behavior. Time in the open arm of the maze is taken to indicate reduced anxiety, an interpretation supported by increased open arm time following administration of anxiolytics such as diazepam (Pellow et al., 1985). We found high open arm time in TBI groups, regardless of housing, possibly due to increased risk-taking behavior after TBI. This is supported by the literature as frontal lobe injury is associated with increased risk-taking behavior in humans (Bechara et al., 1994; Floden et al., 2008). Furthermore, experimental TBI in rats increases the percentage of time spent in the open arms of the EPM (Cutler et al., 2006; Pandey et al., 2009). TBI may result in anxiolytic properties due to damage to the prefrontal cortex. We observed that the effect of injury on open arm time was not abolished by pre-enrichment. The differences between the TBI and Sham groups confirm frontal cortex injury through a gross measurement of behavior. There was no change in EPM activity among groups, confirming that time in open arm may be related to “risk assessment” rather than motor hyperactivity. To further examine anxiety-like behaviors, ethological measures were assessed including head dipping, rearing, and grooming. TBI animals engaged in more head dipping, but this could be attributed to the increased opportunity for dips with increased open arm time. There were no differences in rearing and grooming, supporting the argument that the increased time spent in the open arms is a measure of risk-taking behavior, not anxiety.

The primary goal of this study was to compare the impact of prior housing regimens on the response to injury. The enriched cohort was therefore exposed to a TBI. An ideal design would also include an EC-Sham group. This group would have facilitated the comparison of identical housing conditions, thus elucidating the benefit of pre-EE for injured animals. It is currently not known if non-injured enriched animals perform better than pre-enriched injured animals. Nevertheless, the behavioral results from the current experimental design indicate a neuroprotective benefit of EE before injury. These finding are in line with the large quantities of therapeutic research concluding EE is beneficial after injury. However, the mechanisms governing EE-mediated behavioral improvements after injury are unclear. Behavioral deficits can be attributed to neuronal loss following TBI due to (1) cell death due to physical damage (2) necrotic cell death due to excitatory neurotransmitter release and (3) delayed cell death. It is generally hypothesized that EE applied immediately after injury attenuates delayed cell death. We hypothesize that this is also true when EE is applied before injury, by priming neural processes that promote growth and cell survival.

In summary, the results of this study indicate that EE, when applied before injury, is protective against the functional deficits of TBI. The EE concept may therefore be a viable training mechanism to improve resiliency against the consequences of physical brain trauma. Future studies are necessary to further explore the possible neuroplastic mechanisms responsible for the protection. Understanding how prior environment affects susceptibility to lasting damage from trauma is crucial for protecting at-risk populations (e.g., deployed military personnel) from potentially devastating effects of head injury on sensory and cognitive function.

### Conflict of interest statement

The authors declare that the research was conducted in the absence of any commercial or financial relationships that could be construed as a potential conflict of interest.
